# Susceptibility of *Anopheles sinensis* to *Plasmodium vivax* in malarial outbreak areas of central China

**DOI:** 10.1186/1756-3305-6-176

**Published:** 2013-06-14

**Authors:** Guoding Zhu, Hui Xia, Huayun Zhou, Julin Li, Feng Lu, Yaobao Liu, Jun Cao, Qi Gao, Jetsumon Sattabongkot

**Affiliations:** 1 Key Laboratory of Parasitic Disease Control and Prevention, Ministry of Health, Jiangsu Institute of Parasitic Diseases, Wuxi, Jiangsu Province, China; 2 Jiangsu Provincial Key Laboratory of Parasite Molecular Biology, Jiangsu Institute of Parasitic Diseases, Wuxi, Jiangsu Province, China; 3 Department of Parasitology, Medical College of Soochow University, Suzhou 215123, China; 4 Department of Parasitology, Bengbu Medical College, 2600 Donghai Dadao Road, Bengbu 233030, China; 5 Mahidol Vivax Research Unit, Faculty of Tropical Medicine, Mahidol University, Bangkok, Thailand

**Keywords:** Membrane blood feeding, *Plasmodium vivax*, *Anopheles sinensis*, *Anopheles anthropophagus*

## Abstract

**Background:**

*Anopheles sinensis*, *Anopheles anthropophagus*, *Anopheles minimus* and *Anopheles dirus* are the major vectors of malaria transmission in China. *Anopheles sinensis* is considered a secondary vector due to its relatively low malaria-transmission ability. However, in 2005, an outbreak of over 40,000 *Plasmodium vivax* malaria cases was reported in areas where *Anopheles sinensis* was the only major vector. Therefore, it is necessary to reassess the malaria transmission ability of this vector species in China.

**Methods:**

Laboratory colonies of *An. sinensis* and *An. anthropophagus*, and first-generation progeny (F1) of *An. sinensis* that had been collected in central China, were infected by direct membrane feeding assay with mono-*vivax* gametocyte-containing blood collected from *vivax*-infected patients. The mosquitoes were kept for 7 to 14 days post-blood feeding to allow parasites to develop into oocysts and sporozoites. Infectivity was measured by dissecting midguts and salivary glands. The presence of oocysts and sporozoites was determined by microscopy at 7 and 14 days post-blood feeding, and the numbers of gametocytes and asexual parasites, as well as mosquito parasite infections, were determined.

**Results:**

The positive oocyst and sporozoite feed rates of the 142 pairs of lab-colony *An. sinensis* and *An. anthropophagus* were not significantly different, and the same results were found with the 10 pairs of laboratory and F1 *An. sinensis*. *An. sinensis* had more oocysts/midgut at 7 days post-feeding than *An. anthropophagus*, but the gametocytemia, asexual parasitemia, and ratio of macrogametocytes to microgametocytes, did not correlate with either oocyst or sporozoite infection. However, in the oocyst-positive mosquitoes, there was a correlation between gametocytemia and the average oocyst number/midgut.

**Conclusions:**

The susceptibility of *An. sinensis* (both laboratory and F1) to *P. vivax*-infected blood is similar to *Anopheles anthropophagus*, when evaluated by membrane feeding assay under laboratory conditions. In recent years, in central China, the *vivax* malaria transmission ability of *An. sinensis* has probably been underestimated. Further studies of this species in other regions are needed. *An. sinensis* could also be a good candidate vector for evaluating candidate malaria transmission-blocking vaccines (TBV).

## Background

*Anopheles sinensis*, *An. anthropophagus*, *An. minimus,* and *An. dirus* are the major vectors of malaria transmission in China [1], and species in the *An. maculatus* complex may be major transmission vectors in Tibet Autonomous Region [2]. Among these species, *An. minimus* and *An. dirus* are mostly distributed in southern China (Yunnan and Hainan provinces), where the geographical environment is markedly different from the central region. *An. sinensis* and *An. anthropophagus* are relatively more widely distributed in China. According to the updated distribution records of the *An. hyrcanus* species group (Diptera: Culicidae), *An. sinensis* is found in over 29 provinces and regions in China [3]. It is noteworthy that *An. sinensis* has become the only major vector in central China, where *Plasmodium vivax* is the only prevalent, locally transmitted, malaria parasite; however, a few imported *falciparum* malaria cases have been reported among travellers [4].

*An. sinensis* and *An. anthropophagus* are both members of the *An. hyrcanus* complex, sharing similar morphological characteristics, and a ribosomal DNA-internal transcribed spacer 2 (rDNA-ITS2) -based method is required to distinguish the two species [5,6]. However, in addition to the distinct distributions of *An. sinensis* and *anthropophagus,* the species differ strongly in host preference, resting habitat, and other features involved in malaria transmission. First, *An. anthropophagus* prefers to bite humans rather than animals, whereas *An. sinensis* is a more zoophilic mosquito and demonstrates a marked preference for cattle and other warm-blooded animals. Second, *An. anthropophagus* prefers indoor resting after blood feeding; residual insecticide spraying in areas of central China where *An. anthropophagus* predominated as the major vector for *falciparum* effectively reduced malaria mortality and morbidity, from 1980 to 1990. As a result, *falciparum* malaria has been eliminated in central China. However, *An. sinensis* tends toward outdoor resting after indoor blood feeding, which has made vector control of this species more difficult. Third, *An. anthropophagus* is much more susceptible to *vivax* malaria parasites [7]. The regions in China containing both *An. anthropophagus* and *An. sinensis* have suffered more serious malaria epidemics than those areas where *An. sinensis* is the only vector [8].

Taking the above-mentioned factors into consideration, one possible conclusion is that *An. sinensis* plays a less important role in malaria transmission than other species in central China. Unexpectedly, frequent outbreaks of *vivax* malaria started appearing in areas where *An. sinensis* was the main vector, with over 40,000 reported *vivax* cases in 2005, accounting for 67% of all cases in China [9]; this suggests that the susceptibility and other features of *An. sinensis* that affect its interaction with *vivax* parasites have changed. Thus, the comparative malaria-transmission ability of *An. sinensis* with other major vectors should be reassessed. In this study, we assessed the susceptibility of *An. sinensis* to *P. vivax* in central China by membrane-feeding assay and compared the results with *An. anthropophagus* and a field strain of *An. sinensis*. This study will help to explain the *vivax* epidemic situation in central China better, and improve the current elimination programmes of this species in China.

## Methods

### Study site and patients

**Figure 1:**
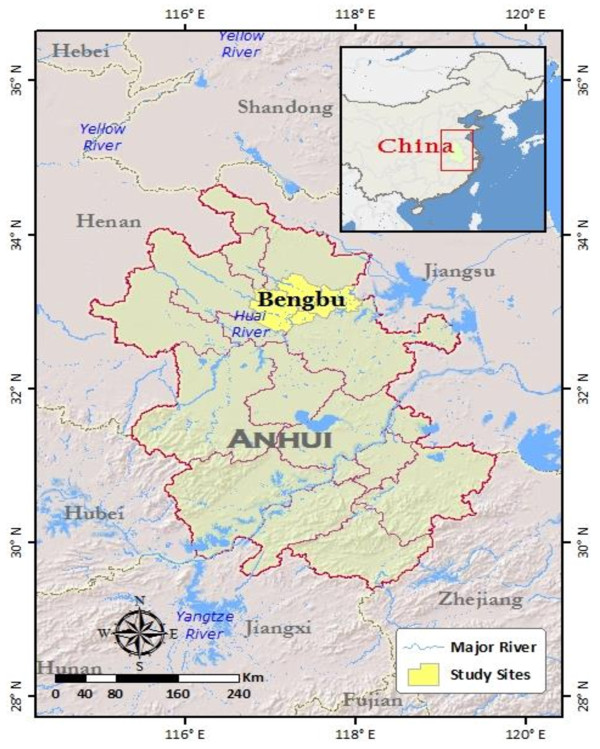
**Study area for *vivax *malaria patient recruitment from 2005 to 2007.**

**Figure 2:**
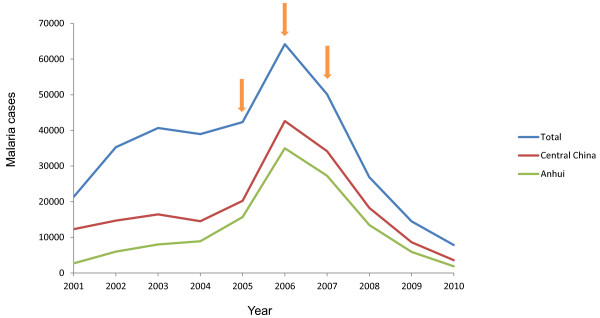
Malaria transmission in China from 2003 to 2010.

The study was conducted in Bengbu, Anhui Province, central China (Figure 1). *P. vivax* is the only malaria parasite in this region. In 2004, the total number of malaria cases in Anhui reached 8,909, which was 22.9% of all cases in China (Figure 2). Patients aged 18 years of age or more, who sought clinical treatment for malaria, were included in this study. Thick and thin blood smears were prepared from each individual and stained with 10% Giemsa by experienced microscopists to exclude mixed infection with *P. falciparum*. In addition, gametocyte and asexual parasite densities were determined for all *P. vivax*-positive patients by counting the number of parasites per 500 leukocytes in a thick blood smear under oil immersion microscopy. The raw counts were converted into parasites/microliter by assuming a count of 8,000 leukocytes/μL. If gametocytes were present, the patient was asked to enrol in the study. After the patients were briefed on the project and completed consent forms, approximately 5 mL of blood was collected by venepuncture and used for membrane blood feeding (detailed below) of starved mosquitoes. After mosquito feeding, the volunteers were released from the study and received antimalarial treatment.

### Mosquitoes

*An. sinensis* and *An. anthropophagus* have been maintained in the insectary of the Key Technical Laboratory for Prevention and Control of Parasitic Diseases of the Ministry of Health (MOH) in the Jiangsu Institute of Parasitic Diseases (JIPD), Wuxi, China, for over 30 years. The mosquitoes were reared at 27 ± 1°C with a relative humidity of 70–80% and provided with 10% (w/v) glucose in water. One hundred mosquitoes in each carrying cage were transported from JIPD to the field sites in a cooler box. Next, 6- to 8-day-old mosquitoes were provided only water for 12 hours prior to blood feeding. In this study, engorged female anopheline mosquitoes from Bengbu, Anhui, were also collected, and their progeny (F1) were identified via both morphological characteristics and an rDNA ITS2-based method [5] to confirm species. The mosquitoes were maintained as described above, prior to blood feeding.

### Membrane feeding

Five hundred microliters (500 μL) of whole blood from each patient were centrifuged at 5,000 g for 5 minutes. The serum was then removed and replaced with approximately 300 μL of AB serum from a *P. vivax-*naive donor. The packed red blood cells and donor sera were carefully mixed and added to the membrane feeder. A constant-temperature (37°C) circulating-water system was used to prevent exflagellation of microgametocytes [10]. The blood feeding lasted for 30 minutes, after which the glass membrane feeder was removed from the top of the carton and all the unengorged mosquitoes were removed and freeze-killed. After feeding, all engorged mosquitoes were transported back to JIPD’s insectary in Wuxi, where they were provided with a 12-h light/dark cycle and daily sugar solution before dissection.

### Mosquito dissection

On day 7 post-blood feeding, the mosquitoes were aspirated into glass tubes and immobilized by placing the tube on ice. At least 10 midguts of each species were dissected in a drop of mercurochrome in phosphate-buffered saline, and the number of oocysts per midgut was counted under 10× or 40× microscopic examination. On day 14 post-feeding, if the mosquitoes were oocyst-positive, another 10 mosquitoes of each species were dissected, and the number of oocysts per midgut was first counted, as above. Then, the infecting sporozoite level was recorded after direct determination under phase contrast microscopy (Leica DM2500, US) without staining. The level was recorded as follows: “+”, 1–10 sporozoites; “++”, 11–100 sporozoites; “+++”, 101–500 sporozoites and “++++”, >500 sporozoites.

### Statistical analysis

The chi-square test was used to compare the proportion of mosquitoes infected with oocysts, the proportion of mosquitoes infected with sporozoites, and the proportion of infected mosquitoes per positive feeding, between paired lab-colony *An. anthropophagus* and *An. sinensis*, and between paired F1 and lab-colony *An. sinensis*. Paired T tests were used to compare oocyst loads (mean oocyst number per infected midgut) between the feeding groups. A regression test was used to detect any linear correlation between parasite load and infection rate.

### Ethical approval

All human-subject research conducted in this study was reviewed and approved by the Institutional Ethics Committee of the National Institute for Parasitic Diseases (NIPD), Chinese Center for Disease Control and Prevention. All the patients involved in this study read and signed the informed consent forms.

## Results

### Patient data

From 2005 to 2007, over 200 symptomatic malaria patients came to the clinic in Bengbu, Anhui. In total, 142 volunteers were finally enrolled in this study after excluding subjects aged less than 18 years and those with mixed infections with *falciparum* malaria, or zero gametocytes by thick blood-smear count. Patient age and parasite density data are shown in Table 1.

**Table 1:** Age and parasite density of the study patients (n = 142)

	**Minimum**	**Maximum**	**Average**
Age of patients (years)	18	71	34.6 ± 3.1
No. of asexual parasites (/μL)	0	21696	4332.8 ± 371.2
No. of macrogametocytes (/μL)	32	6560	1057.6 ± 88
No. of microgametocytes (/μL)	64	4800	486.4 ± 51.2

### Membrane feeding

**Figure 3:**
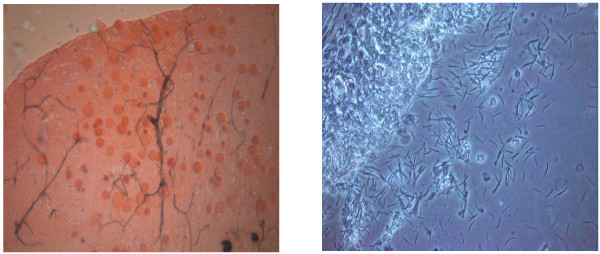
**Oocyst and sporozoite infection in midgut and salivary glands.** (**A**) Oocysts in the midgut were counted using a normal microscope at 10× objective magnification with mercurochrome staining. (**B**) Sporozoites in the salivary glands were assessed using 40× objective magnification and a phase-contrast microscope without staining.

**Figure 4:**
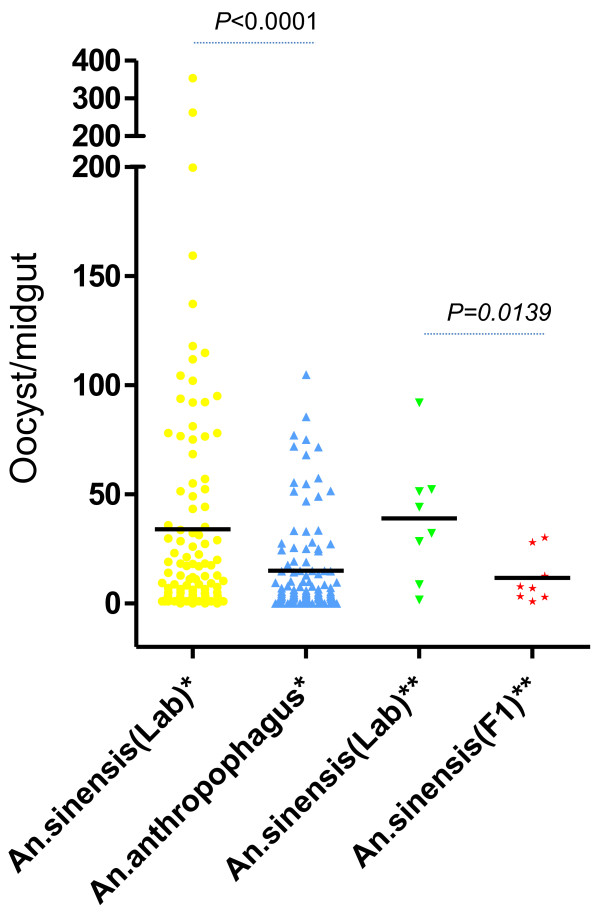
**Scatter plots of the results of the species comparisons.** The median oocyst load of *Anopheles* is shown by a horizontal black line; *: range of oocysts/midgut between the 142 pairs of lab-colony *An. sinensis* (lab) and *An. anthropophagus*, **: range of oocysts/midgut between the 10 pairs of lab-colony (Lab) and first-generation (F1) *An. sinensis*.

In total, the blood of 142 *vivax* patients was fed via membrane feeding to the laboratory colonies of *An. sinensis* and *An. anthropophagus*. Among these 142 patients, blood from 10 patients was also fed to lab-colony and F1 *An. sinensis* mosquitoes. The engorged feeding percentages of the paired laboratory strain *An. sinensis* and *An. anthropophagus* were 64.86% (9210/14200) and 62.86% (8926/14200), respectively. The F1 *An. sinensis* had the lowest engorged feeding rate, at 16.5% (165/1000) (χ2 = 934.05, p < 0.01). Oocyst number and sporozoite index were counted and recorded using a normal and phase-contrast microscope, respectively (Figure 3). The positive oocyst feed rate (positive feeds/total feeds) and positive mosquito infection rate (positive mosquitoes/total mosquitoes) of the lab-colony *An. sinensis* and *An. anthropophagus* did not differ at day 7 post-blood feeding (χ2 = 0.82, P > 0.05, χ2 = 3.22, P > 0.05, respectively). Likewise, the positive sporozoite feed rate and positive mosquito infection rate at day 14 post-blood feeding did not differ (χ2 = 0.09, P > 0.05, χ2 = 0.21, P > 0.05, respectively) (Table 2). In 10 paired cases, both the F1 and laboratory strain had the same positive oocyst feed (80%) and sporozoite feed (30%) rates. In the 10 paired membrane feeding tests, the lab-strain *An. sinensis* had a higher oocyst infection rate at day 7 than F1 (Figure 4), as did the laboratory strain *An. sinensis* in the 142 paired feedings with laboratory strain *An. anthropophagus* (Figure 4). However, interestingly, the two laboratory strains had similar sporozoite levels at day 14 post-feeding (z = 0.866, *p* = 0.38, Table 3).

**Table 2:** **Comparison of blood feeding, and oocyst and sporozoite infection, for*An. sinensis *and *An. anthropophagus***

**Species**	**% of feeds infecting mosquitoes**		**% of mosquitoes that fed on all infectious patients that developed parasite infection**		**Mean number of oocysts per positive mosquito**
	**(Positive feeds/total feeds)**		**(Positive mosquitoes/total mosquitoes)**		
Days post-feeding	7	14	7	14	7
*An. sinensis* (Lab)	72.5 (103/142)	28.9 (41/142)	45.7 (15536/340)	11.1 (135/1216)	45.7 (15536/340)
*An. sinensis* (F1)	80.0 (8/10)	30.0 (3/10)	13.4 (281/21)	20.0 (9/45)	13.4 (281/21)
*An. anthropophagus* (Lab)	67.6 (96/142)	26.8 (38/142)	21.0 (6437/306)	11.84 (96/811)	21.0 (6437/306)

**Table 3:** **Sporozoite infection of *An. sinensis *and *An. anthropophagus *14 days’ post-feeding**

**Sporozoite infective level**	**No. of sporozoites**	
	***An. sinensis*(Lab)**	***An. anthropophagus***
+	12	10
++	42	24
+++	31	19
++++	50	43
Total	135	96

*: “+”, 1–10 sporozoites; “++”, 11–100 sporozoites; “+++”, 101–500 sporozoites and “++++”, >500 sporozoites.

### Correlation of parasitemia and infection

**Figure 5:**
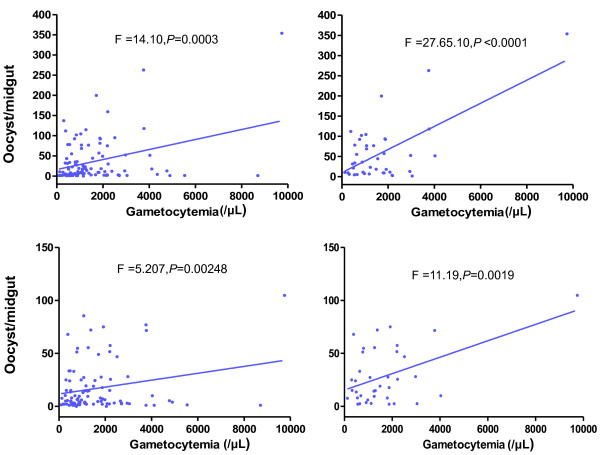
**Correlation between number of *Plasmodium vivax *gametocytes per microliter of blood and number of oocysts per midgut. ** (**A**) Lab-colony *An. sinensis* (103 oocyst-positive cases), (**B**) Lab-colony *An. sinensis* (41 sporozoite-positive cases), (**C**) Lab-colony *An. anthropophagus* (96 oocyst-positive cases) and (**D**) Lab-colony *An. anthropophagus* (38 sporozoite-positive cases).

In total, 41/103 and 38/96 infected lab-colony *An. sinensis* and *An. anthropophagus*, respectively, developed sporozoites in the salivary glands at 14 days’ post-blood feeding. The other 62 and 58 respective cases only had oocysts in the midgut at day 7 post-feeding, and 32 of 142 cases were negative for both oocyst and sporozoite infection in both *An. sinensis* and *An. anthropophagus* (Table 4). The effects of parasite density (macrogametocyte, microgametocyte, asexual-stage parasite) and ratio of macrogametocytes to microgametocytes, in the five groups referred to above, were evaluated. The parasite density or the ratio of macrogametocyte to microgametocyte had no effect on parasite infection as the data showed that there was no significant difference between the negative and positive cases. However, in cases of positive infection, regression analysis revealed a significant linear correlation between blood gametocyte density and midgut parasite infection load in both *An. sinensis* and *An. anthropophagus.* The cases with more oocysts or sporozoites had higher gametocytemia levels, particularly in the sporozoite-positive cases (Figure 5).

**Table 4:** **Parasitemia and oocyst and sporozoite infection in *An. sinensis *and *An. anthropophagus***

**Species**	***An. sinensis***		***An. anthropophagus***		***An. sinensis/An. anthropophagus***
	**Oocyst(+)/ Sporozoite(−)**	**Oocyst(+)/ Sporozoite(+)**	**Oocyst(+)/ Sporozoite(−)**	**Oocyst(+)/ Sporozoite(+)**	**Oocyst(−)/ Sporozoite(−)**
Cases	62	41	58	38	32
Mean oocysts/midgut	20.81	58.03	18.36	53.68	0
Mean gametocyte density (/μL)	1579.6 ± 154.8	1682.3 ± 255.4	1618.2 ± 163.4	1652.2 ± 267.9	1386.5 ± 254.0
Mean asexual parasite density (/μL)	4308.2 ± 444.4	5061.8 ± 709.6	4325.0 ± 456.6	4329.7 ± 663.2	3781.5 ± 714.2
Female gametocytes/male gametocytes	3.24 ± 0.25	3.41 ± 0.46	3.31 ± 0.26	3.07 ± 0.35	3.98 ± 0.92

## Discussion

This is the first study to evaluate the susceptibility of *An. sinensis* to *vivax* parasites in central China by membrane feeding, after the re-emergence of malaria in central China. In this study, *An. sinensis* (both laboratory colony and F1) were equally susceptible to *vivax* malaria parasites as *An. anthropophagus,* which was believed for many years to be the major vector in central China. Despite that belief, the laboratory colony of *An. sinensis* had a higher oocyst infection rate. Although the same was not found in the F1 mosquitoes, their low observed *vivax* susceptibility could have been due to their low engorged feeding rate, caused by the switch of emergent environment from field to laboratory. In addition, the difficulty of maintaining the engorged mosquitoes under laboratory conditions cannot be ignored [11], as this definitely reduced the quantity of mosquitoes dissected at day 7 post-feeding. Furthermore, in the 10 paired cases, both laboratory and field *An. sinensis* mosquitoes presented the same infection rate and 100% concordance with the positive case selection (both positive oocyst and sporozoite feedings), suggesting the lab-colony *An. sinensis* in this study could well represent actual current vector susceptibility to parasites in the field. In this case, *An. sinensis* was more able to carry *P. vivax* in the midgut stage than *An. anthropophagus,* given an equal opportunity to feed on malaria patients. This contrasts with previous results in central China [12]. Indeed, only mosquitoes with sporozoites in the salivary glands can infect humans, although both *An. sinensis* and *An. anthropophagus* had similarly low sporozoite infection rates in this study, which should raise some suspicion [13]. However, it is reasonable to note that malaria parasites also reduce mosquito survival rates [14]. In addition, our objective was to evaluate the susceptibility of *An. sinensis* compared with *An. anthropophagus* and not to count sporozoite quantities long-term. Both mosquito species had more sporozoites after 14 days’ post-feeding than at 14 days. Thus, oocyst infection in the midgut stage could reflect potential transmission capacity.

A well-known preference for human biting, a tendency to rest indoors, and great susceptibility to parasites with sufficient longevity, are essential criteria for evaluating vectors for malaria transmission capacity. In China, although *An. sinensis* is the most widely distributed, with a large population in most mainland regions, the species had for decades been judged not to be the predominant vector for malaria due to its exophilic and exophagic features, and relatively low susceptibility to parasites compared with other vectors. Nevertheless, the *vivax* malaria outbreak in 2005 in central China, in which *An. sinensis* served as the main vector, suggested an updated evaluation of the vector capacity and transmission role of this species was necessary [15]. Along with agricultural and industrial progress in China, frequently moving populations have become an important group at risk of carrying parasites from malaria-epidemic areas to malaria-free or low-transmission regions. During the malaria transmission season, from June to September, farmers and construction workers habitually sleep in the open without net protection, which increases the chance for *An. sinensis*, which in this study had a strong propensity to develop *vivax* malaria parasites following blood-feeding from infected humans, to bite several different people. Due to the exophilic nature of *An. sinensis* and continuously increasing insecticide resistance [16-21], the regular insecticide residual spray (IRS) methods used in malaria-transmission regions do not kill all mosquitoes [22]. Another possible reason for the malaria outbreak in central China is climatic and environmental change [10]. If *An. sinensis* mosquitoes are unable to find their usual animal blood feeding targets, because of the construction of buildings or other such changes, they may resort to biting humans [23].

Only macrogametocytes and microgametocytes can develop in the mosquito midgut; all other asexual parasites are digested after blood feeding. This study supports the previous finding that although the average asexual parasitemia counts in the negative feed groups were lower than the positive feed groups, no significant differences were found [24]. This was also supported by some positive feeds with zero asexual parasites somehow achieving a high oocyst infection number; however, the oocysts were absent in the midgut in some cases with high asexual parasite counts. Although the same phenomenon was noted with the relationship of gametocytemia to midgut infection in the negative and positive groups, oocyst load (oocysts per positive mosquito) increased with gametocyte density in all infection-positive groups, suggesting that the blood of patients with high levels of gametocytemia had a greater potential to induce mosquitoes, post-feeding, to develop oocysts in the midgut and thereby be at higher risk of transferring parasites to other humans. Although the results from this study confirm the previous finding that oocysts developed well in the mosquito midgut, with a ratio of macrogametocytes to microgametocytes of less than 4 [25], the number of negative-infection cases producing oocysts in this study demonstrated that the presence of oocysts in the midgut following feeding is not a good indicator of infectivity, a conclusion supported by a study in west Thailand [26]. The malaria parasite is under intense attack from the mosquito’s innate immune system during its development in the midgut and salivary glands [27]. Several mosquito immune genes play important roles in the parasite evasion stage by influencing parasite-mosquito interactions [28-30]. In other words, the susceptibility of mosquitoes to malaria infection could be related to an enhanced or weakened immune response of mosquitoes to parasite infection [31]. Additionally, the genotypes of the invading parasites play an important role, i.e., parasites with VK210 and VK247, two main genotypes of circumsporozoite protein (CSP), have an obvious preference for infecting mosquitoes [32]. Therefore, further study of the susceptibility of *An. sinensis* to parasites from various geographic areas in China is necessary.

Although the direct-feeding method more accurately reflects epidemiologic reality [33], most volunteers prefer to provide blood by venepuncture rather than allowing mosquitoes to bite their skin directly [34]. In the membrane-feeding assay used in this study, patient sera were replaced by naive malaria-free human AB serum, to avoid interference from varying antibody levels in patient blood samples [35]. Furthermore, the constant-temperature cycling system allowed unlimited maintenance of parasite activity and equalised blood-feeding conditions among the mosquito groups [36]. The membrane feeding assay is a valuable tool for the evaluation and validation of candidate markers of transmission-blocking vaccine (TBV) following the modification of target genes [37,38]. Because *An. sinensis* is the largest of the four major vectors in China, as well being relatively easier to maintain under laboratory conditions, and with high susceptibility to *vivax* parasites, it could be used as a valuable candidate species to evaluate TBV, in particular.

Compared to the previous studies [39,40] the *An sinensis* from this study showed higher susceptibility rates to *P. vivax* isolates in Central China, although it is known that the *An. sinensis* strain from Korea, China and Japan was compatible genetically and/or nearly identical to that from Thailand, based on the crossing experiments and comparative sequence analyses of the ribosomal DNA (rDNA) internal transcribed spacer 2 (ITS2) [41]. The genetic diversity of the parasites and their compatibility to the vectors in each location may contribute to the difference in vector susceptibility. In this study we did not analyse the genetic diversity of the parasites as the study aimed to compare the susceptibility of *An. sinensis* and *An. anthopophagus* in Central China to the same parasite isolates collected in this region.

## Conclusions

To our knowledge, this is the first report of the susceptibility of the widely distributed malaria vector *An. sinensis* to *P. vivax* following artificial membrane feeding after the re-emergence of malaria in central China. The *An. sinensis* mosquitoes in the laboratory maintained a similar capacity to become infected with *vivax* parasites as the field mosquitoes, and their parasite-carrying ability was also similar to that of *An. anthropophagus*. The vector capacity of *An. sinensis* for malaria transmission during the *vivax* re-emergence period, particularly in central China, has probably been underestimated. Due to its morphological characteristics and high susceptibility to parasites, *An. sinensis* could be a good vector candidate for *vivax* malaria TBV evaluation.

## Competing interests

The authors declare that they have no competing interests.

## Authors’ contributions

GD, QG, and JS contributed equally to the study design and data analysis. HX, HY, JL, FL, YB and JC managed the parasites and mosquitoes in the field. GD, HY, JL and YB contributed to the dissection of mosquitoes. GD drafted the manuscript. QG provided scientific supervision. JS revised the manuscript. All authors read and approved the final manuscript.
